# Dl-3-n-butylphthalide protects the heart against ischemic injury and H9c2 cardiomyoblasts against oxidative stress: involvement of mitochondrial function and biogenesis

**DOI:** 10.1186/s12929-017-0345-9

**Published:** 2017-06-15

**Authors:** Xiaochao Tian, Weiliang He, Rong Yang, Yingping Liu

**Affiliations:** 1 Department of Cardiology, The Second Hospital of Hebei Medical University, Shijiazhuang, Hebei 050000 People’s Republic of China; 2 Department of Neurology, Hebei General Hospital, Shijiazhuang, Hebei 050000 China; 3 Department of Cardiology, Beijing Shijitan Hospital, Capital Medical University, Beijing, 100038 China

**Keywords:** Myocardial infarction, Dl-3-n-butylphthalide, Mitochondrial function, Mitochondrial biogenesis

## Abstract

**Background:**

Myocardial infarction (MI) is an acute and fatal condition that threatens human health. Dl-3-n-butylphthalide (NBP) has been used for the treatment of acute ischemic stroke. Mitochondria may play a protective role in MI injury. However, there are few reports on the cardioprotective effect of NBP or the potential mitochondrial mechanism for the NBP-induced protection against cardiac ischemia injury. We investigated the therapeutic effects of NBP in an in vivo MI model and an in vitro oxidative stress model, as well as the potential mitochondrial mechanism.

**Methods:**

This study comprised two different experiments. The aim of experiment 1 was to determine the protective effects of NBP on MI and the underlying mechanisms in vivo. In part 1, myocardial infarct size was measured by staining with 2,3,5-triphenyltetrazoliumchloride (TTC). Myocardial enzymes and mitochondrial enzymes were assayed. The aim of experiment 2 was to investigate the role of NBP in H_2_O_2_-induced myocardial ischemic injury in H9c2 cells and to determine the potential mechanism. In part 2, H9c2 cell viability was evaluated. ROS levels, mitochondrial morphology, and mitochondrial membrane potential of H9c2 cells were measured. ATP levels were evaluated using an assay kit; mitochondrial DNA (mtDNA), the expressions of NRF-1 and TFAM, and mitochondrial biogenesis factors were determined.

**Results:**

NBP treatment significantly reduced the infarct ratio, as observed by TTC staining, decreased serum myocardial enzymes in MI, and restored heart mitochondrial enzymes (isocitrate dehydrogenase (ICDH), succinate dehydrogenase (SDH), malate dehydrogenase (MDH), and a-ketoglutarate dehydrogenase (a-KGDH) activities after MI. Moreover, in in vitro studies, NBP significantly increased the viability of H9c2 cells in a dose-dependent manner, reduced cell apoptosis, protected mitochondrial functions, elevated the cellular ATP levels, and promoted H_2_O_2_-induced mitochondrial biogenesis in H9c2 cardiomyoblasts.

**Conclusion:**

Collectively, the results from both the in vivo and in vitro experiments suggested that NBP exerted a cardioprotective effect on cardiac ischemic injury via the regulation of mitochondrial function and biogenesis.

## Background

Myocardial infarction (MI), the most common cardiovascular complaint, is an acute and fatal condition of the cardiovascular system that threatens human health [1]. The ischemic heart experiences cardiomyocyte loss, with subsequent reparative fibrotic healing, decreased cardiac function, which plays an important role in progression of cardiac hypertrophy, and arrhythmia [2]. Although myocardial protection has recently improved, there are still a limited number of effective therapies to improve myocardial resistance to ischemic injury [3]. Thus, the identification of novel drug targets for the treatment of MI is imperative.

The abundance of mitochondria in the heart is well known; they are known shown to provide continuous energy. It has become increasingly clear that mitochondrial function is involved in ischemic injury. Impaired mitochondria disturb ATP generation [4]. A close association has been identified between altered mitochondrial morphology and dynamics and MI injury [5]. It has also been reported that the protection of mitochondria in the post-infarct myocardium resulted in decreased mitochondrial dysfunction and cardiomyocyte apoptosis [6]. In addition, oxidative stress occurred mainly in the mitochondria; damaged cells attack the mitochondria [7]. Researchers have shown that oxidative stress plays an important role in myocardial ischemic injury [8, 9]. Ischemic oxidative stress results in the reduction of myocardial antioxidants, loss of mitochondrial membrane potential, and release of superoxide [10, 11]. Therefore, the identification of a pharmacological agent that exerts protective effects in mitochondria and alleviates MI injury might be an ideal cardioprotective strategy.

Dl-3-n-butylphthalide (NBP), a small molecule extracted from a Chinese herb (Chinese celery), has been approved for the treatment of acute ischemic stroke by the State Food and Drug Administration of China [12]. Previous studies have shown that NBP conferred neuroprotective effects via the improvement of microcirculation dysfunction during ischemia [13], decreased the cerebral infarct area in brain ischemia models [14], protected neuron activity in stroke [15], and attenuated inflammatory responses [16] in cultured astrocyte models. In addition, NBP has preventive and therapeutic effects on the improvement of outcomes after cardiac arrest and resuscitation [17]. Recent studies have indicated that NBP ameliorated oxidative stress and mitochondrial damage, which reduced endothelial cell death after oxygen glucose deprivation in vitro [18]. It has been reported that oxidative stress affected the mitochondrial apoptotic pathway [19]. However, at present, the effect of NBP on mitochondrial function and energy metabolism in MI have not been comprehensively described.

Therefore, the aims of this study were to investigate the therapeutic effects of NBP on in vivo MI models, as well as to determine the potential mechanism with a focus on the mitochondrion in vitro oxidative stress models.

## Methods

### Animals and ethical statement

The experimental protocols used in this study were in accordance with the National Institutes of Health Guide for Care and Use of Laboratory Animals. All animal manipulations were performed in accordance with the recommendations of the Committee of the Care and Use of Laboratory Animals at Hebei Medical University.

### Preparation of rat MI model

Rats were anesthetized by the intraperitoneal injection of chloral hydrate (300 mg/kg), intubated, and ventilated using a small-animal ventilator (Ardmore PA, USA) with a tidal volume of 12 mL/kg. After a left thoracotomy at the fourth intercostal space, the heart was exposed, the pericardium was opened, and the left anterior descending coronary artery (LAD) was ligated. Rats in the sham group underwent the same surgical procedures, with the exception that the LAD was not ligated, in accordance with previous reports [1].

### Experimental protocols

**Fig. 1:**
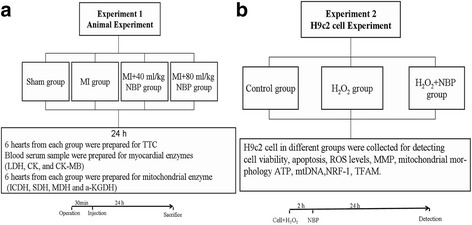
Experiment design. In experiment 1 **a**, animals were divided into four groups: Sham, MI, 40 mg/kg NBP, and 80 mg/kg NBP groups. Rats in the NBP-treated groups were injected intraperitoneally with 40 mg/kg NBP and 80 mg/kg NBP at 30 min after MI. The infarct area, cardiac enzymes, and mitochondrial enzyme were measured 24 h after MI. In experiment 2 **b**, H9c2 cardiomyoblasts were divided into three groups: control, H_2_O_2_, and H_2_O_2_ + NBP 10 μM groups. The cells were pre-treated with 100 μM H_2_O_2_ for 2 h and subsequently treated with NBP for 24 h. Then, cell viability, apoptosis, mitochondrial function, and biogenesis were detected

As shown in Fig. 1, this study was divided into two experiments. The aim of experiment 1 was to determine the protective effects of NBP on MI and the underlying in vivo mechanism. The rats were assigned randomly into four groups: Sham, MI, MI + 40 mg/kg NBP, and MI + 80 mg/kg NBP. Rats in the 40 mg/kg MI-induced groups underwent MI and were then injected intraperitoneally with 40 mg/kg NBP and 80 mg/kg NBP, respectively, at 30 min after LAD ligation. After 24 h, the rats were sacrificed and the hearts were stored frozen until further analysis. The aim of experiment 2 was to investigate the role of NBP in H_2_O_2_-induced myocardial ischemic injury in H9c2 cells and to determine the potential mechanism. H_2_O_2_ (100 μM) was used to induce oxidative insult. The cells were pre-treated with 100 μM H_2_O_2_ for 2 h, subsequently with the treatment of NBP for 24 h. Control experiments were performed in the absence of NBP or presence of H_2_O_2_; the H_2_O_2_ injury group was treated with 100 μM H_2_O_2_ for 2 h only.

### Measurement of infarct size

Myocardial infarct size was measured by staining with TTC in accordance with previous reports [20]. At 24 h after surgery, the hearts were sliced into slices of 2 mm thickness, incubated in 1% TTC at 37 °C for 20 min, followed by fixation in 4% paraformaldehyde for 10 min. Image-J software was used to analyze the infarcted area. The results were expressed as a percentage of infarct size by the division of the calculated total infarct size with the total heart volume.

### Myocardial enzyme assays

At 24 h after surgery, serum was separated from the blood by centrifugation at 2500 × *g* for 5 min at 4 °C. The myocardial enzymes (lactate dehydrogenase [LDH], creatine kinase [CK], and MB isoenzyme of creatine kinase [CK-MB]) were measured in accordance with the manufacturer’s instructions (Nanjing Jiancheng Bioengineering Institute, China).

### Measurement of mitochondrial enzyme activity

Mitochondrial enzyme assays were performed as reported previously [21]. The activities of isocitrate dehydrogenase (ICDH), succinate dehydrogenase (SDH), malate dehydrogenase (MDH), and a-ketoglutarate dehydrogenase (a-KGDH) were assayed separately.

### H9c2 cardiomyoblast culture

H9c2 cells derived from rat embryonic cardiomyoblasts were obtained from American Type Culture Collection (ATCC). The cells were cultured in Dulbecco’s modified Eagle’s medium (DMEM) supplemented with 10% fetal bovine serum (FBS) and incubated in a humidified 5% CO_2_ incubator at 37 °C. The medium was changed 2–3 times per week.

### Cell viability assay

To assess the protective potential of NBP against oxidative stress, H9c2 cell viability was evaluated by using the Cell Counting Kit-8 (CCK-8) (Dojindo, Japan). In brief, the cells were seeded in 96-well plates at 1 × 10^5^ cells/well and allowed to adhere for 24 h. The culture medium was removed and different concentrations of NBP were added to the wells. At 24 h after treatment, 10 μL CCK-8 was added and the plates were incubated for an additional 2 h. The optical density (OD) was measured at a wavelength of 450 nm by using a microtiter plate reader (Tecan, Switzerland). The OD values were presented as the percentage change compared with the control. Additionally, H9c2 cell apoptosis was evaluated. For flow cytometry, the cells were detached and labeled using the Annexin V-FITC/PI apoptosis detection kit in accordance with the manufacturer’s instructions. Apoptotic cells were quantified by using a FACSCalibur cytometer (Becton Dickinson, Franklin Lakes, New Jersey, USA).

### Measurement of reactive oxygen species (ROS), mitochondrial morphology, and mitochondrial membrane potential (MMP)

The ROS levels were quantified in accordance with the protocol of the reactive oxygen species assay kit (Beyotime, China). The samples were incubated with DCFH-DA at 37 °C for 45 min in the dark. The samples were then washed three times with PBS. The fluorescence intensity was recorded at 485 nm (excitation) and 535 nm (emission) using a microplate reader (Tecan, Switzerland). Data were presented as a percentage of the control.

Mitochondrial morphology was elucidated by MitoTracker Red CMXRos dye (Molecular Probes, M7512). After drug treatment for 24 h, the living cells were incubated with 50 nmol/L MitoTracker Red for 30 min at 37 °C. After incubation, the cells were washed three times with PBS. Fluorescence images were obtained using the red channels of an upright fluorescence microscope (Olympus, Tokyo, Japan).

The mitochondrial membrane potential (MMP) of H9c2 cells was measured by using the JC-1 assay kit (Beyotime, China). Briefly, after the indicated treatments in 6-well plates, the cells were collected and incubated with a solution of JC-1 stain (5 μg/mL) for 20 min at 37 °C. The cells were then rinsed twice with JC-1 staining buffer and centrifuged at 600 × *g* for 15 min at 4 °C. The cells were suspended in JC-1 staining buffer and the fluorescence intensity of JC-1 was detected by using a monochromator microplate reader (Tecan, Switzerland). The values of the MMP were represented as a percentage change compared with the control. The fluorescence images were obtained using the green channels on an upright fluorescence microscope (Olympus, Tokyo, Japan).

### Detection of cellular ATP levels

The ATP assay kit (Beyotime, China) was employed to detect the cellular ATP levels. Briefly, after the indicated treatments in 6-well plates, the cells were dissociated with cell lysis buffer and centrifuged at 12000 × *g* for 5 min at 4 °C. The supernatant of cells was collected and used. Luminance was measured by a monochromator microplate reader (Tecan, Switzerland). The data were presented as a percentage change compared with the control.

### Mitochondrial DNA Quantification

Total DNA derived from H9c2 cells extracted using the DNeasy Blood and Tissue kit (Qiagen, US) according to the manufacturer’s instructions. mtDNA copy number was measured by real-time PCR method using an ABI 7500 real-time PCR system (Applied Biosystems, Foster, California) with the SYBR Green detection method. The relative mtDNA copy number (mt DNA) was determined by a real-time polymerase chain reaction (qPCR), and compared relative to nuclear DNA (rRNA 18S). The primers for mt DNA were forward, 5′- AACACGATCAGGCAACCAAA -3′, and reverse 5′-GGTAGCGGGTGAGTTGTCAG -3′. Primers for rRNA 18S were forward, 5′-GGACAGCGGGTGAGTTGTCA-3′, and reverse 5′-ACCTTCGTTATCGGA ATACC-3′.

### Western blot analysis

Total proteins of cells were extracted according to the manufacturer’s instructions (Applygen Technologies Inc, Beijing). The concentrations of protein were quantified with the Bicinchoninic Acid Protein Assay (BCA, Thermo Fisher Scientific, USA). Equivalent proteins were loaded by 10% sodium dodecyl sulfate-polyacrylamide gel electrophoresis (SDS-PAGE) and transferred to a PVDF membrane (Bio-Rad Laboratories, USA). The membranes were blocked with 5% nonfat milk solution in tris-buffered saline with 0.1% Triton X-100 (TBST, Invitrogen, Carlsbad, CA, USA) for 1 h, and then incubated with rabbit anti-NRF-1 (1:200, Santa Cruz Biotechnology, USA),or rabbit anti-TFAM (1:200, Santa Cruz Biotechnology, USA) overnight at 4 °C. The membranes were then rinsed with 0.1% TBST and subsequently incubated in 0.1% TBST containing fluorescent labeling second antibodies (IRDye® 800-conjugated rabbit anti-mouse 1:8000 dilution; Rockland, Gilbertsville, PA, USA) for 1 h. Bands were visualized by an enhanced chemiluminescent substrate (Thermo Fisher Scientific, USA). For internal loading control, a rat anti-glyceraldehyde 3-phosphate dehydrogenase (GAPDH) (1:5000, Sigma, USA) was used. Western blot data were quantified by densitometric film scanning and normalized to GAPDH levels.

### Statistical analysis

Statistical analysis was performed by using SPSS version 17.0. All data were presented as the mean ± SEM. Statistical evaluation of the data was performed by one-way ANOVA followed by the Student-Newman-Keuls test for intergroup comparisons. A value of *P < 0.05* was considered statistically significant.

## Results

### NBP reduces infarct ratio and decreases serum myocardial enzymes in MI

**Fig. 2:**
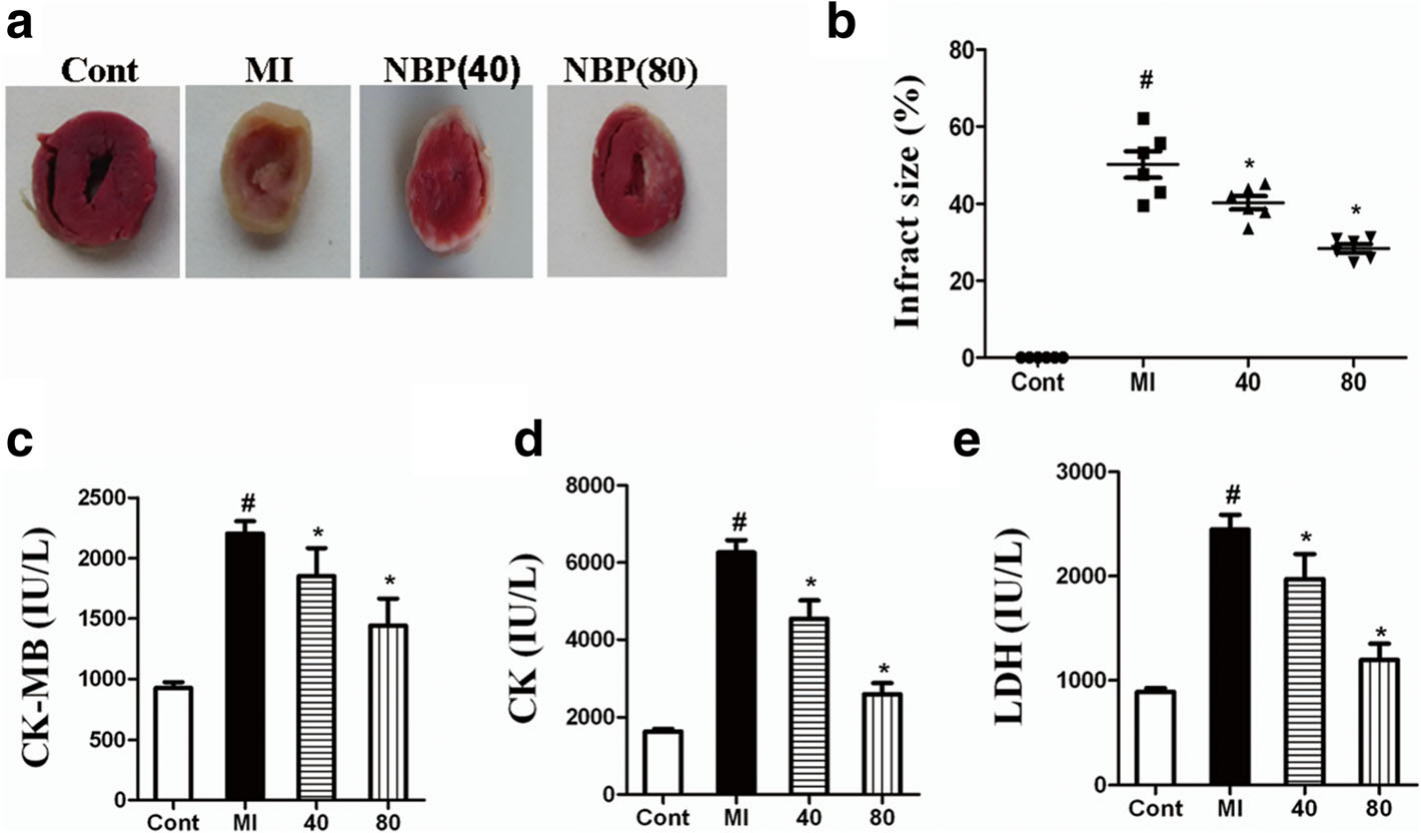
Infarct area and cardiac enzymes in the different groups in experiment 1. At 24 h after MI, the hearts of the rats were collected for the detection of the infarct area and cardiac enzymes. Data revealed that NBP significantly reduced the infarct area of rats following MI **a**, **b**. Moreover, the levels of cardiac enzymes were reduced after NBP treatment **c**-**e**. # *P* < 0.05 vs Sham group; *P < 0.05 vs MI group

First, we assessed whether NBP protected cardiac myocytes in MI. As shown in Fig. 2, no infarction was observed in the sham group and the infarct area increased in the MI group (50.27 ± 8.24%). However, NBP treatment decreased the infarct area in comparison to the MI group *(P < 0.05)*. Significant differences in infarct size were also observed among different NBP-treated groups (40 mL/kg NBP:40.31 ± 4.28%; 80 mL/kg NBP: 28.43 ± 2.73%) (*P < 0.05*).

In addition, the levels of serum myocardial enzymes (CK-MB, CK, and LDH) were significantly increased in rats in the MI group in comparison with the control group (*P < 0.05*). However, after NBP treatment, the serum levels of CK-MB, CK, and LDH were reduced in a dose-dependent manner (40 mL/kg and 80 mL/kg), as shown in Fig. 2b, c, and d. These results revealed that NBP significantly improved the functional recovery after MI.

### NBP restores heart mitochondrial function after MI

**Fig. 3:**
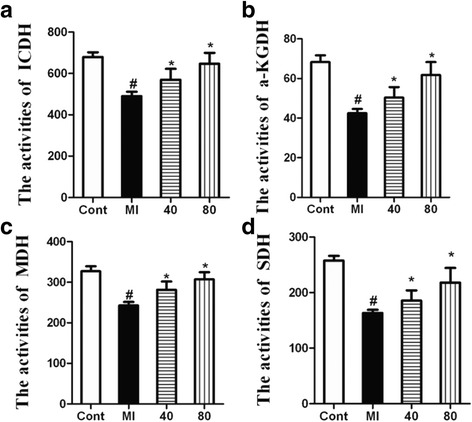
Effect of NBP on mitochondrial enzyme activities in MI. The activity is expressed as nM of NADH oxidized/h/mg protein for ICDH; nM of ferrocyanide formed/h/mg protein for a-KGDH; nM of succinate oxidized/min/mg protein for SDH; and nM of NADH oxidized/min/mg protein for MDH. Values represent the mean ± S.E.M. (*n* = 6). # *P* < 0.05 vs Sham group; *P < 0.05 vs MI group

Mitochondria play a vital role in the supply of energy to the heart. In order to reveal the underlying mechanisms of the protective effect of NBP on the mitochondrion in MI, we evaluated the mitochondrial enzymes. The activities of ICDH, a-KGDH, SDH, and MDH were significantly decreased in untreated heart mitochondria after MI, whereas the activities of ICDH, a-KGDH, SDH, and MDH were markedly restored by NBP treatment (Fig. 3). These results suggested that NBP could effectively restore mitochondrial function in the MI-insulted heart.

### NBP exerts a protective effect on H_2_O_2_-induced oxidative stress in H9c2 cardiomyoblasts in vitro

**Fig. 4:**
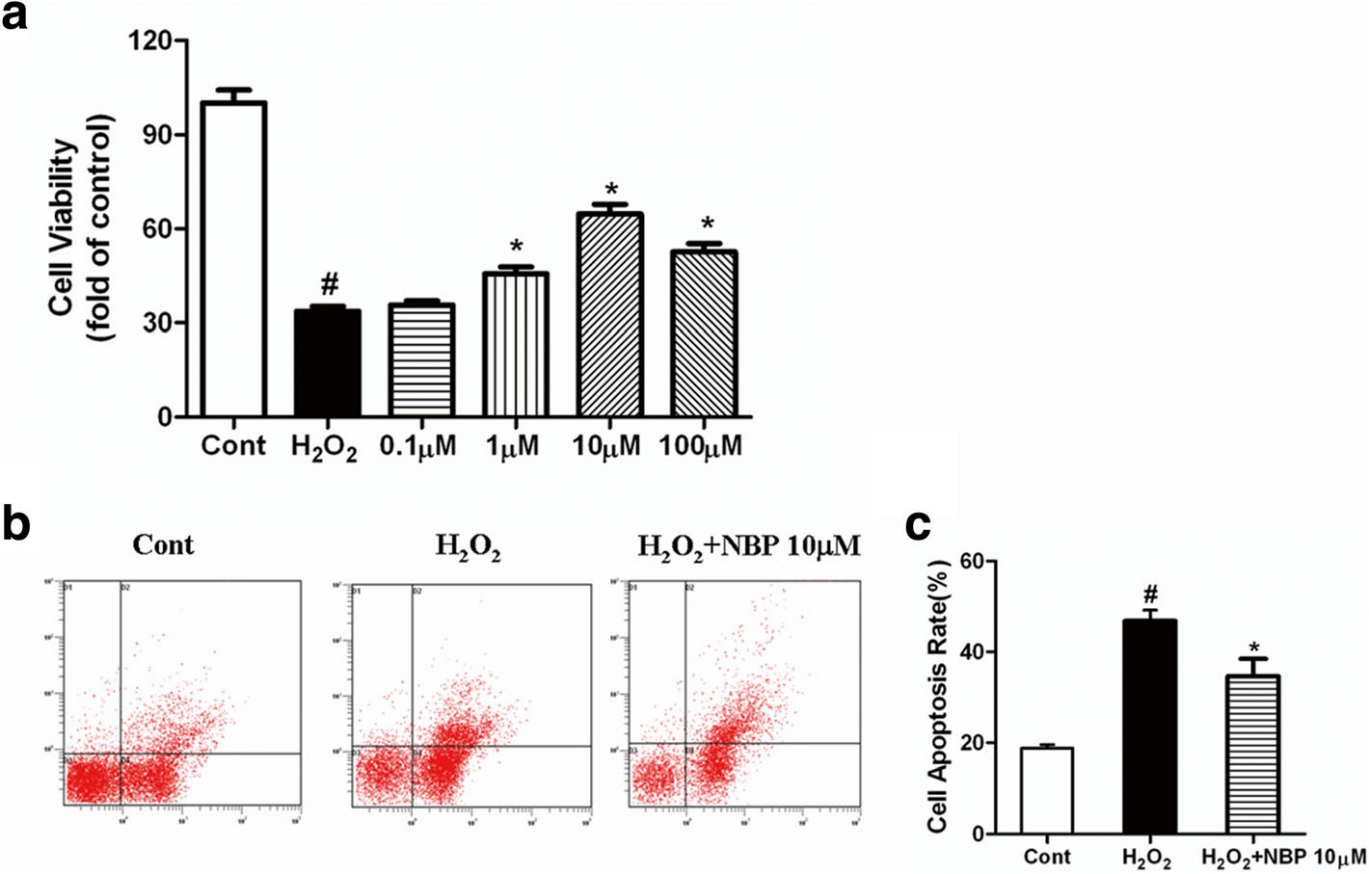
Effect of NBP on cell viability and apoptosis in H9c2 cardiomyoblasts exposed to H_2_O_2_. **a** The cell viability was determined by CCK-8 assay. Value were the mean ± S.E.M, *n* = 6, # *P* < 0.05 vs. Control group; * *P* < 0.05 vs H_2_O_2_ group. **b** The cell apoptosis was determined by Annexin V-FITC/PI staining. Depicted are the mean ± S.E.M, # *P* < 0.05 vs Control group; * *P* < 0.05 vs H_2_O_2_ group

Oxidative stress is the basic pathophysiological origin of the development of myocardial infarction. Therefore, H_2_O_2_-induced H2c9 oxidative stress imitated myocardial ischemia in vitro. To investigate the protective function of NBP after H_2_O_2_-induced insult, cell viability and apoptosis were analyzed. The results of the CCK-8 assay indicated that H_2_O_2_ treatment decreased the cell viability compared with the control group, whereas NBP increased the viability of cells insulted by H_2_O_2_. Moreover, NBP treatment increased the viability of H_2_O_2_-treated cells in a concentration dependent manner (Fig. 4a). Based on the above findings, 10 μM NBP was selected as the best dosage for the subsequent experiments. The percentage of the apoptotic cells was 18.84 ± 1.88, 46.80 ± 5.89, 34.68 ± 3.79 in the control, H_2_O_2_, and H_2_O_2_ + NBP groups, respectively. Flow cytometric analysis also demonstrated that NBP significantly decreased the apoptosis of H9c2 cells treated with H_2_O_2_ (Fig. 4b and c).

### NBP protects mitochondrial functions against oxidative stress in H9c2 cardiomyoblasts

**Fig. 5:**
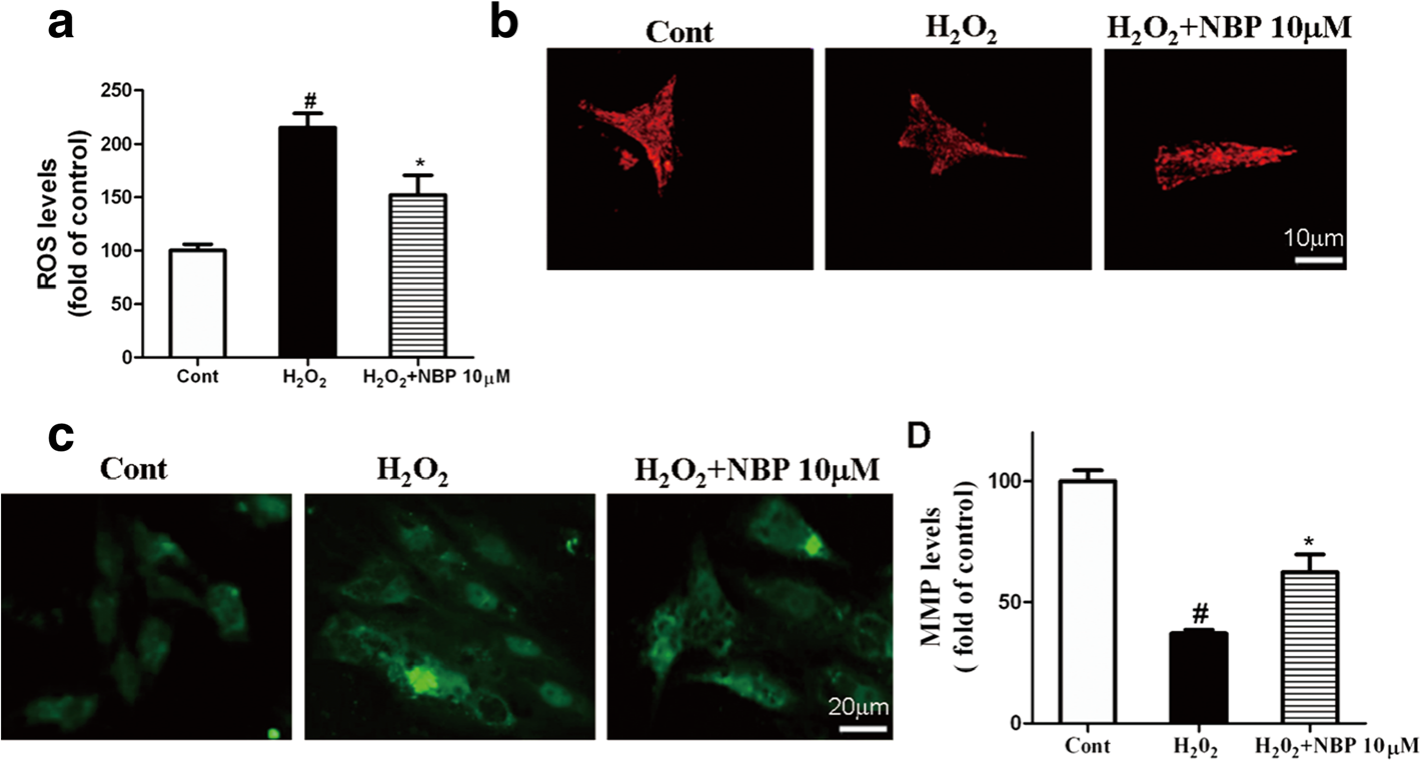
The influence of NBP on mitochondrial function in H9c2 cardiomyoblasts exposed to H_2_O_2_. **a** The generation of ROS in different groups (Control group; H_2_O_2_ group; H_2_O_2_ + NBP group). **b** Micrographs of mitochondrial morphology by MitoTracker Red staining in H9c2 (Control group; H_2_O_2_ group; H_2_O_2_ + NBP group). **c** Mitochondrial membrane potential (MMP) was determined by evaluation of JC-1 staining under an upright fluorescence microscope after use of the JC-1 assay kit. **d** Cells were exposed to H_2_O_2_ for 2 h and then MMP was determined in the absence or presence of NBP. Results were expressed as the mean ± S.E.M. # *P* < 0.05 vs Control group; * *P* < 0.05 vs H_2_O_2_ group

To investigate the underlying mechanism of NBP treatment on mitochondria subjected to oxidative stress in vitro, the fluorescent probe DCFH-DA was used to detect ROS release. Compared with the control, ROS was generation was increased in the H_2_O_2_-treated group. However, NBP significantly inhibited the ROS generation induced by H_2_O_2_, as shown in Fig. 5a. The immunofluorescence results also indicated that, compared with the control group, H_2_O_2_ treatment altered the mitochondrial morphology from elongated to uniformly punctate organelles. However, the co-treatment of NBP and H_2_O_2_ resulted in the elongation of mitochondria (Fig. 5b).

To further ascertain the protective effect of NBP with regard to mitochondrial damage, the MMP, an indicator of mitochondrial function, was measured by the JC-1 assay kit. Quantitative data demonstrated that NBP attenuated the loss of MMP induced by H_2_O_2_ in H9c2 cells (Fig. 5c and d). These findings provided evidence that NBP exerted a protective effect against oxidative stress on the mitochondrial function in H9c2 cells.

### NBP elevates H9c2 cellular ATP levels suppressed by H_2_O_2_

**Fig. 6:**
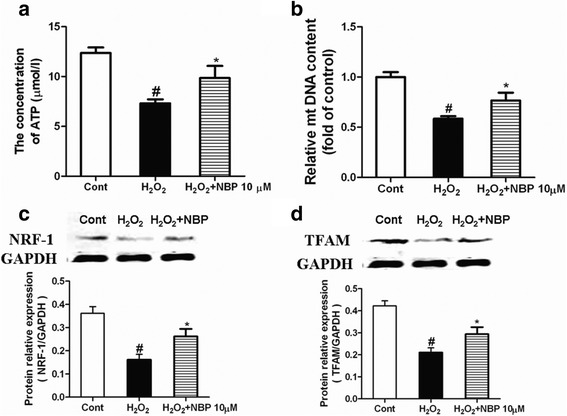
NBP promotes mitochondrial biogenesis after H_2_O_2_ treatment. The mitochondrial ATP generation **a** and mitochondrial DNA (mtDNA) content **b** were measured in different groups. The expression of NRF-1, and TFAM at protein levels was measured by western blot **c** and **d** Data are shown as the mean ± S.E.M. # *P* < 0.05 vs Control group. * *P* < 0.05 vs H_2_O_2_ group

As described above, NBP protected the mitochondrial function of H9c2 cells against oxidative stress. As mitochondria are the main source of energy generation, we examined the ATP levels. NBP significantly elevated the cellular ATP levels that were reduced by exposure to H_2_O_2_ (Fig. 6a). The above results indicated that NBP could increase the ATP levels suppressed by H_2_O_2_ in the vitro model.

### NBP treatment promotes mitochondrial biogenesis of H9c2 cells after H_2_O_2_ insult

To determine whether NBP affected mitochondrial biogenesis, the content of mitochondrial DNA (mtDNA) was measured (Fig. 6b); it was found that H_2_O_2_ treatment significantly decreased the mtDNA content, whereas NBP treatment increased the mtDNA content after H_2_O_2_ treatment. Moreover, we also measured the expressions of NRF-1, and TFAM, mitochondrial biogenesis factors, by using western blotting (Fig. 6c and d). The results showed that NBP significantly increased the protein levels of these factors after the exposure of cells to H_2_O_2_.

## Discussion

MI leads to the irreversible loss of cardiomyocytes, which has been reported as one of the leading causes of morbidity and mortality in China [22]. Therefore, it is particularly vital to explore new drugs for the treatment of MI. Our research provided the novel perspective that dl-3-n-butylphthalide (NBP), a neuroprotective drug used for the treatment of stroke in China, also exerted a cardioprotective effect via the improvement of mitochondrial function and biogenesis in MI.

NBP, a pure component from seeds of *Apium graveolens* Linn., is widely used clinically for the treatment of ischemic stroke [12]. NBP has been reported to exert many anti-ischemic effects on the central neural system [23, 24]. Additionally, NBP has been found to improve motor performance, extend the survival interval, and attenuate motor neuron loss to extend survival by the attenuation of glial activation in a mouse model of amyotrophic lateral sclerosis [25]. NBP protected neuronal cells from MPP(+)-associated cell injury in Parkinson’s disease [26] and significantly delayed the onset and progression of diabetic cataracts [27]. In addition, recent studies revealed that NBP protected VSMC from PDGF-BB-stimulated proliferation. NBP has both preventive and therapeutic effects on the improvement of outcomes after cardiac arrest and resuscitation [17]. NBP protects cardiomyocytes from ischemia/reperfusion-induced apoptosis [19]. However, the effect of NBP on MI remains unclear. In the current study, we investigated the myocardial protective effects of NBP on MI. In experiment 1, our research showed that different concentrations of NBP exerted myocardial protective effects via the reduction of the infarct area and decreased levels of serum myocardial enzymes in a rat model of MI. Oxidative stress is one of the major causative factors of MI. Therefore, we evaluated the viability and apoptosis of H9c2 cells treated with NBP and exposed to oxidative stress. The observations revealed that NBP attenuated H_2_O_2_-induced injury in H9c2 cells in vitro. The above results suggested that NBP exerted a cardioprotective effect on MI.

Mitochondria perform a critical role in the supply of energy to the heart; mitochondrial dysfunction is closely associated with cardiac dysfunction [28]. In order to reveal the underlying mechanisms of the mitochondrial dysfunction in MI, researchers have mainly focused on mitochondrial impairment, especially in mitochondrial enzymes, during MI [29–31]. The protection of mitochondrial enzymes in MI may improve the energy metabolism and cardiac functional recovery [32]. NBP suppressed mitochondrial dysfunction to reduce the cytotoxicity of MPP(+) in Parkinson’s disease [26]. NBP protects oxygen glucose deprivation-induced endothelial cells against mitochondrial damage in vitro [18]. However, the effect of NBP on the activities of mitochondrial enzymes in MI has not been reported. In experiment 1 of this study, we observed that NBP treatment increased the activities of ICDH, a-KGDH, SDH, and MDH during MI, which suggested that NBP probably protected the myocardium from MI by the regulation of the mitochondrial enzyme activities.

Oxidative stress is the basic pathophysiological process responsible for the development of myocardial infarction. Oxidative stress induces the overaccumulation of intracellular ROS, which initiates cellular apoptosis and causes myocardial damage. ROS produced mainly in the mitochondria, but damaged cells occur after mitochondrial attack [7]. The preservation of mitochondrial functions, including the maintenance of the mitochondrial membrane potential and a decrease in ROS generation, may be beneficial in the treatment of myocardial ischemic injury. It was previously reported that antioxidants protected H9c2 cardiomyoblasts against hydrogen peroxide-mediated disruption of the mitochondrial membrane permeability transition [33]. Owing to its antioxidative properties, NBP protected bone marrow stem cells against apoptosis induced by oxidative stress [34]. NBP protects dopaminergic neurons, most likely by maintaining mitochondrial membrane potential and preventing the generation of reactive oxygen species (ROS) [35]. In our study, we evaluated the protective effect of NBP on the mitochondrial function of myocardial cells exposed to oxidative stress. Our results showed that NBP treatment decreased ROS activity, improve impaired mitochondrial morphology, and attenuated the loss of H_2_O_2_-induced MMP in H9c2 cells. These results suggested that NBP treatment prevented changes in ROS generation, mitochondrial morphology, and MMP in H9c2 cells exposed to oxidative stress.

Mitochondrial function plays a prominent role in the induction of apoptotic cell death after oxidative stress [36]. Increased mitochondrial function can result from increased mitochondrial biogenesis [37]. The most important function of mitochondrial biogenesis is mitochondrial homeostasis, which maintains mitochondrial function [38]. Mitochondrial biogenesis has been found to enhance the attenuation of the detrimental consequences of oxidative stress and has been suggested as a novel component of the repair mechanism [39]. Impaired mitochondrial biogenesis is a central mechanism responsible for myocardial injury following MI [40]. The decrease in mitochondrial biogenesis is a key component of the development of heart failure after MI [41]. It has been reported that mitochondrial biogenesis was severely impaired in MI and that the preservation of mtDNA copy number alleviated impaired mitochondrial biogenesis [42]. Our results showed that NBP-treatment could protect mitochondrial function in H9c2 cells exposed to oxidative stress. Thus, we hypothesized that there might be a subtle link between NBP and mitochondrial homeostasis in MI. In our study, we detected the total amount of intact mtDNA. Our results showed that NBP-treatment restored the mtDNA loss induced by H_2_O_2_ in H9c2 cells. NRF-1 has been demonstrated to modulate energy supply and control mitochondrial biogenesis [43]. TFAM is also a key molecule in the maintenance of mtDNA and the regulation of copy number [44]. We tested the protein levels of NRF-1 and TFAM of H9c2 cells exposed to oxidative stress. The results indicated that NBP treatment resulted in the restoration of mtDNA loss induced by H_2_O_2_ in H9c2 cells. Collectively, the data indicate that the NBP-induced cardioprotection against oxidative stress was partially mediated through enhanced mitochondrial biogenesis.

## Conclusion

In conclusion, the current findings demonstrated that treatment with NBP exerted cardioprotective effects on MI via the regulation of mitochondrial enzymes. In vitro studies on H9c2 cardiomyoblasts confirmed the cardioprotective effect of NBP occurred via the regulation of mitochondrial function and biogenesis. Collectively, the study suggested that further pharmacological evaluation of NBP might be helpful to identify potential for therapeutic interventions for MI that target mitochondria.

## References

[1] Chen O, Ye Z, Cao Z, Manaenko A, Ning K, Zhai X, Zhang R, Zhang T, Chen X, Liu W, Sun X (2016). Methane attenuates myocardial ischemia injury in rats through anti-oxidative, anti-apoptotic and anti-inflammatory actions. Free Radic Biol Med..

[2] Shyu KG, Wang BW, Cheng WP, Lo HM (2015). MicroRNA-208a increases myocardial endoglin expression and myocardial fibrosis in acute myocardial infarction. Can J Cardiol.

[3] Pei H, Song X, Peng C, Tan Y, Li Y, Li X, Ma S, Wang Q, Huang R, Yang D, Li D, Gao E, Yang Y (2015). TNF-α inhibitor protects against myocardial ischemia/reperfusion injury via Notch1-mediated suppression of oxidative/nitrative stress. Free Radic Biol Med..

[4] Chan PH (2004). Mitochondria and neuronal death/survival signaling pathways in cerebral ischemia. Neurochem Res..

[5] Yang XM, Cui L, White J, Kuck J, Ruchko MV, Wilson GL, Alexeyev M, Gillespie MN, Downey JM, Cohen MV (2015). Mitochondrially targeted Endonuclease III has a powerful anti-infarct effect in an in vivo rat model of myocardial ischemia/reperfusion. Basic Res Cardiol.

[6] Wang F, Yang J, Sun J, Dong Y, Zhao H, Shi H, Fu L (2015). Testosterone replacement attenuates mitochondrial damage in a rat model of myocardial infarction. J Endocrinol.

[7] Cao G, Minami M, Pei W (2001). Intracellular Bax translocation after transient cerebral ischemia: implications for a role of the mitochondrial apoptotic signaling pathway in ischemic neuronal death. J Cereb Blood Flow Metab.

[8] Aldakkak M, Camara AK, Heisner JS, Yang M, Stowe DF (2011). Ranolazine reduces Ca2+ overload and oxidative stress and improves mitochondrial integrity to protect against ischemia reperfusion injury in isolated hearts. Pharmacol Res.

[9] Montecucco F, Lenglet S, Braunersreuther V, Pelli G, Pellieux C, Montessuit C, Lerch R, Deruaz M, Proudfoot AE, Mach F (2010). Single administration of the CXC chemokine-binding protein Evasin-3 during ischemia prevents myocardial reperfusion injury in mice. Arterioscler Thromb Vasc Biol.

[10] Hansen SH, Andersen ML, Cornett C, Gradinaru R, Grunnet N (2010). A role for taurine in mitochondrial function. J Biomed Sci.

[11] Yang Y, Zhang Y, Liu X, Zuo J, Wang K, Liu W, Ge J (2013). Exogenous taurine attenuates mitochondrial oxidative stress and endoplasmic reticulum stress in rat cardiomyocytes. Acta Biochim Biophys Sin (Shanghai).

[12] Cui LY, Zhu YC, Gao S, Wang JM, Peng B, Ni J, Zhou LX, He J, Ma XQ (2013). Ninety-day administration of dl-3-n-butylphthalide for acute ischemic stroke: a randomized, double-blind trial. Chin Med J (Engl).

[13] Chang Q, Wang XL (2003). Effects of chiral 3-n-butylphthalide on apoptosis induced by transient focal cerebral ischemia in rats. Acta Pharmacol Sin.

[14] Peng Y, Sun J, Hon S, Nylander AN, Xia W, Feng Y, Wang X, Lemere CA (2010). L-3-n-butylphthalide improves cognitive impairment and reduces amyloid-beta in a transgenic model of Alzheimer’s disease. J Neurosci.

[15] Ji XC, Zhao WH, Cao DX, Shi QQ, Wang XL (2011). Novel neuroprotectant chiral 3-n-butylphthalide inhibits tandem-pore-domain potassium channel TREK-1. Acta Pharmacol Sin.

[16] Wang HM, Zhang T, Huang JK, Sun XJ (2013). 3-N-butylphthalide (NBP) attenuates the amyloid-β-induced inflammatory responses in cultured astrocytes via the nuclear factor-κB signaling pathway. Cell Physiol Biochem.

[17] Zhang L, Puchowicz MA, LaManna JC, Xu K (2016). Protective Effect of Dl-3-n-Butylphthalide on recovery from cardiac arrest and resuscitation in rats. Adv Exp Med Biol..

[18] Li L, Zhang B, Tao Y, Wang Y, Wei H, Zhao J, Huang R, Pei Z (2009). DL-3-n-butylphthalide protects endothelial cells against oxidative/nitrosative stress, mitochondrial damage and subsequent cell death after oxygen glucose deprivation in vitro. Brain Res..

[19] Wang YG, Li Y, Wang CY, Ai JW, Dong XY, Huang HY, Feng ZY, Pan YM, Lin Y, Wang BX, Yao LL (2014). L-3-n-Butylphthalide protects rats’ cardiomyocytes from ischaemia/reperfusion-induced apoptosis by affecting the mitochondrial apoptosis pathway. Acta Physiol (Oxf).

[20] Miller EJ, Li J, Leng L, McDonald C, Atsumi T, Bucala R, Young LH (2008). Macrophage migration inhibitory factor stimulates AMP-activated protein kinase in the ischaemic heart. Nature.

[21] Aristatile B, Al-Numair KS, Al-Assaf AH, Pugalendi KV (2011). Pharmacological effect of carvacrol on D: -galactosamine-induced mitochondrial enzymes and DNA damage by single-cell gel electrophoresis. J Nat Med.

[22] Zhang C, Sun A, Zhang S, Yao K, Wu C, Fu M, Wang K, Zou Y, Ge J (2010). Efficacy and safety of intracoronary autologous bone marrow-derived cell transplantation in patients with acute myocardial infarction: insights from randomized controlled trials with 12 or more months follow-up. Clin Cardiol.

[23] Wei W, Zhang W, Huang Y, Li Y, Zhu G, Chen F, Li J (2012). The therapeutic effect of (DL)-3-n-butylphthalide in rats with chronic cerebral hypoperfusion through downregulation of amyloid precursor protein and matrix metalloproteinase-2. J Int Med Res.

[24] Xue LX, Zhang T, Zhao YW, Geng Z, Chen JJ, Chen H (2016). Efficacy and safety comparison of DL-3-n-butylphthalide and Cerebrolysin: effects on neurological and behavioral outcomes in acute ischemic stroke. Exp Ther Med.

[25] Feng X, Peng Y, Liu M, Cui L (2012). DL-3-n-butylphthalide extends survival by attenuating glial activation in a mouse model of amyotrophic lateral sclerosis. Neuropharmacology.

[26] Huang JZ, Chen YZ, Su M, Zheng HF, Yang YP, Chen J, Liu CF (2010). dl-3-n-Butylphthalide prevents oxidative damage and reduces mitochondrial dysfunction in an MPP(+)-induced cellular model of Parkinson’s disease. Neurosci Lett.

[27] Wang F, Ma J, Han F, Guo X, Meng L, Sun Y, Jin C, Duan H, Li H, Peng Y (2016). DL-3-n-butylphthalide delays the onset and progression of diabetic cataract by inhibiting oxidative stress in rat diabetic model. Sci Rep..

[28] Silambarasan T, Manivannan J, Priya MK, Suganya N, Chatterjee S, Raja B (2015). Sinapic acid protects heart against ischemia/reperfusion injury and H9c2 cardiomyoblast cells against oxidative stress. Biochem Biophys Res Commun.

[29] Ikeuchi M, Matsusaka H, Kang D, Matsushima S, Ide T, Kubota T, Fujiwara T, Hamasaki N, Takeshita A, Sunagawa K, Tsutsui H (2005). Overexpression of mitochondrial transcription factor a ameliorates mitochondrial deficiencies and cardiac failure after myocardial infarction. Circulation.

[30] Yu P, Zhang J, Yu S, Luo Z, Hua F, Yuan L, Zhou Z, Liu Q, Du X, Chen S, Zhang L, Xu G (2015). Protective effect of sevoflurane postconditioning against cardiac ischemia/reperfusion injury via ameliorating mitochondrial impairment, Oxidative Stress and Rescuing Autophagic Clearance. PLoS One.

[31] Dodd MS, Atherton HJ, Carr CA, Stuckey DJ, West JA, Griffin JL, Radda GK, Clarke K, Heather LC, Tyler DJ (2014). Impaired in vivo mitochondrial Krebs cycle activity after myocardial infarction assessed using hyperpolarized magnetic resonance spectroscopy. Circ Cardiovasc Imaging.

[32] Higuchi T, Miyagawa S, Pearson JT, Fukushima S, Saito A, Tsuchimochi H, Sonobe T, Fujii Y, Yagi N, Astolfo A, Shirai M, Sawa Y (2015). Functional and electrical integration of induced pluripotent stem cell-derived cardiomyocytes in a myocardial infarction rat heart. Cell Transplant.

[33] Park C, So HS, Shin CH, Baek SH, Moon BS, Shin SH, Lee HS, Lee DW, Park R (2003). Quercetin protects the hydrogen peroxide-induced apoptosis via inhibition of mitochondrial dysfunction in H9c2 cardiomyoblast cells. Biochem Pharmacol.

[34] Sun B, Feng M, Tian X, Lu X, Zhang Y, Ke X, Huang S, Cao J, Ding X (2012). DL-3-n-Butylphthalide protects rat bone marrow stem cells against hydrogen peroxide-induced cell death through antioxidation and activation of PI3K-Akt pathway. Neurosci Lett.

[35] Xiong N, Huang J, Chen C, Zhao Y, Zhang Z, Jia M, Zhang Z, Hou L, Yang H, Cao X, Liang Z, Zhang Y, Sun S, Lin Z, Wang T (2012). Dl-3-n-butylphthalide, a natural antioxidant, protects dopamine neurons in rotenone models for Parkinson’s disease. Neurobiol Aging.

[36] Verdin E, Hirschey MD, Finley LW, Haigis MC (2010). Sirtuin regulation of mitochondria: energy production, apoptosis, and signaling. Trends Biochem Sci.

[37] Cheng A, Hou Y, Mattson MP (2010). Mitochondria and neuroplasticity. ASN Neuro.

[38] Seo AY, Joseph AM, Dutta D, Hwang JC, Aris JP, Leeuwenburgh C (2010). New insights into the role of mitochondria in aging: mitochondrial dynamics and more. J Cell Sci.

[39] McLeod CJ, Pagel I, Sack MN (2005). The mitochondrial biogenesis regulatory program in cardiac adaptation to ischemia--a putative target for therapeutic intervention. Trends Cardiovasc Med.

[40] Tao L, Bei Y, Lin S, Zhang H, Zhou Y, Jiang J, Chen P, Shen S, Xiao J, Li X (2015). Exercise training protects against acute myocardial infarction via improving myocardial energy metabolism and mitochondrial biogenesis. Cell Physiol Biochem.

[41] Powers SK, Smuder AJ, Kavazis AN, Quindry JC (2014). Mechanisms of exercise-induced cardioprotection. Physiology (Bethesda).

[42] Inoue T, Ikeda M, Ide T, Fujino T, Matsuo Y, Arai S, Saku K, Sunagawa K (2016). Twinkle overexpression prevents cardiac rupture after myocardial infarction by alleviating impaired mitochondrial biogenesis. Am J Physiol Heart Circ Physiol.

[43] McLeod CJ, Jeyabalan AP, Minners JO, Clevenger R, Hoyt RF, Sack MN (2004). Delayed ischemic preconditioning activates nuclear-encoded electron-transfer-chain gene expression in parallel with enhanced postanoxic mitochondrial respiratory recovery. Circulation.

[44] Kang D, Hamasaki N (2005). Mitochondrial transcription factor A in the maintenance of mitochondrial DNA: overview of its multiple roles. Ann N Y Acad Sci..

